# Late Metastasis in Conjunctival Adenosquamous Carcinoma

**DOI:** 10.18502/jovr.v19i3.10084

**Published:** 2024-09-16

**Authors:** Hassan Lami, Sarrvesa Hari Vijay Singh, Svetlana Cherepanoff, J. Males

**Affiliations:** ^1^Faculty of Medicine, University of New South Wales, Sydney, Australia; ^2^Pathology/Sydpath, St Vincent's Hospital, Sydney, Australia; ^3^Faculty of Medicine, University of Sydney, Sydney, Australia; ^5^Hassan Lami: https://orcid.org/0000-0002-4376-011X

**Keywords:** Adenosquamous Carcinoma, Lymph Node Metastasis, Metastatic Adenosquamous Carcinoma

## Abstract

**Purpose:**

To present a rare case of metastatic conjunctival adenosquamous carcinoma (ASC) in the context of limited literature on the prognosis of ASC and suggested follow-up and surveillance.

**Case Report:**

We report a case of conjunctival ASC that metastasized to cervical lymph nodes five years after histological confirmation of complete local excision.

**Conclusion:**

Long-term clinical follow-up and surveillance imaging are warranted to allow early detection of disease recurrence and/or metastasis.

##  INTRODUCTION

Conjunctival adenosquamous carcinoma (ASC) is a very rare tumor composed of two cell types, squamous and glandular, that mostly affects males in their 60s.^[1]^ ASC is distinct from mucoepidermoid carcinoma (MEC) of conjunctiva in several ways:

(i) unlike MEC, ASC is typically associated with carcinoma in situ of the surface epithelium; (ii) invasive ASC appears more squamous superficially, with the glandular “adeno’’ component best seen at the infiltrating base of the tumor; (iii) MEC consists of a mix of three distinct cells types – epidermoid, mucous, and intermediate, whereas ASC consists of two cells types – those with true squamous differentiation and gland forming epithelial cells; and (iv) unlike MEC, ASC is not associated with translocations involving the MAML gene.

Recently, there has been a debate on the existence of primary MEC of conjunctiva, since early classification systems depended on morphology only and some cases of ASC may have been misclassified as MEC.^[1]^ However, due to the rarity of both MEC and ASC, there is as yet insufficient published data to rebut MEC as a subtype of primary conjunctival carcinoma. Conjunctival ASC is known to be highly aggressive locally, with only a few reported metastatic cases.^[2]^ Here, we report a case of conjunctival ASC that metastasized to cervical lymph nodes five years following histological confirmation of complete local excision.

##  CASE REPORT

A 62-year-old Caucasian male was referred to a cornea specialist in March 2016 by his optometrist due to a suspicious conjunctival mass. There was no history of ocular pathology.

**Figure 1:**
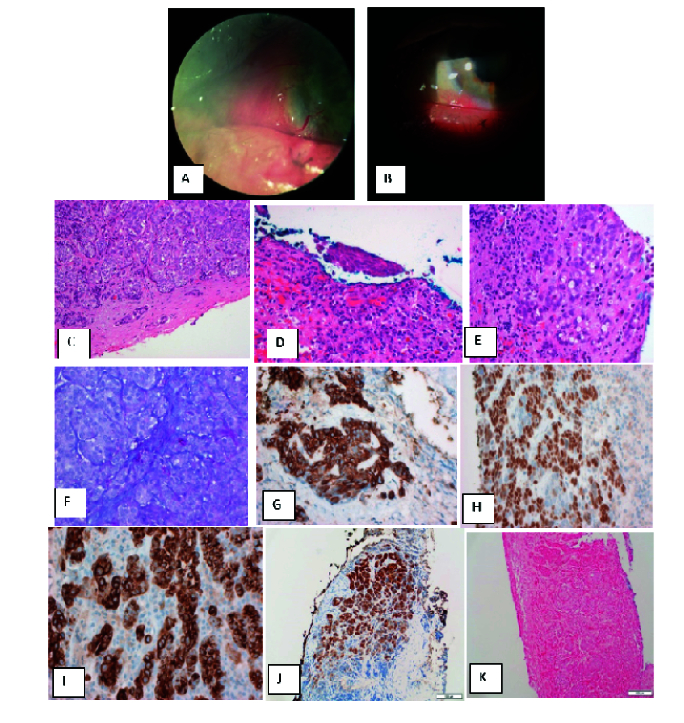
(A) Slit-lamp picture of conjunctival ASC before surgical excision. (B) Slit-lamp picture of conjunctival ASC after second surgical excision with a 7-mm lamellar corneal transplant. (C) H&E staining ( × 400). (D) H&E staining ( × 200). (E) Alcian Blue/DPAS ( × 400). (F) H&E staining ( × 400). (G) CK5/6 ( × 400). (H) p63 ( × 400). (I) CK7 ( × 400). (J) p16 ( × 200). (K) HPV ISH ( × 200).

The first clinical examination revealed an area of left-sided corneal thinning and a possible melt that was associated with pterygium and was clinically suspicious for squamous cell carcinoma [Figure 1A]. The affected area was treated with excision of the pterygium and the suspicious limbal area, followed with cryotherapy. Histopathology of the limbal lesion showed ulcerated conjunctiva and a basophilic, nested tumor infiltrating substantia propria [Figures 1C & 1D]. Tumor nests showed focal glandular differentiation, with DPAS-positive and Alcian-blue negative epithelial mucin [Figure 1E]. Squamous dysplasia was seen in the minimally-intact surface conjunctiva [Figure 1F]. An immunohistochemical panel showed positive CK5/6, p63, BerEP4, EMA, and CK7 immunostaining, along with focal adipophilin staining; however, MART1, GATA3, synaptophysin, chromogranin, bcl-2, and TTF1 were negative [Table 1; Figures 1G–1K]. Furthermore, P16 was positive, although HPV in situ hybridization (Ventana Inform family III high risk probe) was negative [Figure 1I]. No lymphovascular or perineural invasion was seen.

A deeper dissection of the left limbal lesion revealed that ASC had extended to the margins of excision, with no evidence of lymphovascular or perineural invasion.

Informed by the histopathology, a further 7-mm diameter sclero-corneal excision and double freeze-thaw cryotherapy were performed within the base of the lesion. This excision was free of tumor on histological assessment. Subsequently, a 7-mm lamellar corneal transplantation was performed as a tectonic procedure [Figure 1B]. Local radiotherapy was not performed due to the negative re-excision and to avoid the risk of corneal thinning and perforation. The patient was regularly followed-up by his local ophthalmologist every six months.

In early 2021, the patient was diagnosed with cervical lymphadenopathy at routine ophthalmology follow-up. Fine needle aspiration biopsy of the level-1 left cervical lymph node showed poorly differentiated carcinoma. The left neck dissection confirmed a 13-mm deposit of metastatic carcinoma in a level-1 (submental) lymph node. There was nearly complete lymph node replacement by carcinoma, which was arranged in sheets and showed central necrosis. No extranodal extension was seen. Level-2 and -3 cervical lymph nodes were negative. Immunohistochemistry performed on the metastatic deposit showed an immunoprofile similar to the primary conjunctival ASC [Table 1]. Due to morphological and immunohistochemical compatibility with the primary ASC, the metastatic deposit was considered of conjunctival origin. The patient did not have a history of other malignancies. Clinical examination and FDG PET/CT imaging did not reveal other possible primary tumor sites. The patient is under clinical surveillance and is currently disease-free.

This case report adhered to the tenets of the Declaration of Helsinki. The patient involved in this case report has been de-identified. For this reason, written informed consent was not obtained and this was approved by the ethics committee. This study protocol was reviewed and approved by St Vincent’s Human Research Ethics Committee approval Ref. 201//236.

**Table 1:** Immunohistochemistry results from 2016 and 2021.


	**Primary conjunctival ASC (2016)**	**Metastatic carcinoma; submental lymph node (2021)**
CK 5/6	Positive	Positive
CK 7	Positive	Positive
P63	Positive	Weak and Focal
EMA	Positive	Scattered
BerEP4	Positive	Weak and Focal
Adipophilin	Positive (focal)	Negative
GATA3	Negative	•
MART1	Negative	•
Bcl-2	Negative	•
Synaptophysin	Negative	Negative
Chromogranin	Negative	Negative
P16	Positive	Positive
TTF-1	Negative	Positive (patchy)
CK 20	•	Negative
P40	•	Negative
CDX2	•	Negative
PSA	•	Negative
NAPSIN A	•	Negative
CD56	•	Negative
INSM1	•	Negative
NUT	•	Negative
SMA	•	Negative
Calponin	•	Negative
INI –1	•	Retained
	
	

##  DISCUSSION

This case adds to the limited literature on metastatic conjunctival ASC which usually affects organs such as the stomach, pancreas, and intestines.^[3]^ ASC is known to be very aggressive with a high risk of metastasis.^[3]^ While multiple case reports have documented extraocular ASC metastasis to the conjunctiva, there is a considerably lower rate of primary conjunctival ASC metastasis to regional lymph nodes.^[2]^ Kase et al reported the first known case of ASC in the conjunctiva of a 76-year-old female in 2014.^[4]^ She was initially diagnosed with squamous cell carcinoma and was treated with radiotherapy. However, due to the rapid reoccurrence of the tumor, she underwent orbital exenteration and was subsequently diagnosed with ASC.^[4]^ At the time of the report (13 years after the initial presentation), the patient did not have any local recurrence or distant metastases.^[4]^ A more recent case of ASC has been a 79-year-old male presented with metastases in the periparotid lymph node.^[5]^ The patient had positive excision margins and was lost to follow-up for seven months before MMC 0.4% was administered. This case differs from our patient in terms of age and negative margins, further highlighting the importance of close monitoring in ASC follow- up.^[5]^

To the best of our knowledge, this is the first published case in Australia documenting nodal metastasis five years after the excision of primary conjunctival ASC.

The treatment of conjunctival carcinoma depends on the stage of the cancer as described in the eighth edition of the AJCC manual, in addition to the extent of ocular involvement, lymph node involvement, and distant metastasis.^[3]^ Due to its aggressive nature, propensity for local invasion, and high rate of recurrence, the mainstay of treatment for conjunctival ASC is complete surgical excision.^[2,3]^ In cases of intraocular involvement, early enucleation is recommended to minimize metastatic risk or the need for later exenteration.^[3]^ Topical IFN α -2b and mitomycin C have been suggested as adjunctive treatments after local resection of conjunctival ASC to reduce the risk of local recurrence or metastasis.^[2,6]^ Considering the aggressive nature of ASC, it is recommended to determine the clinical stage of the condition by performing PET/CT imaging of the head, neck, chest, abdomen, and pelvis in order to exclude regional and distant metastasis.

In summary, this report documents primary metastatic conjunctival ASC five years after complete surgical excision, suggesting that long-term clinical follow-up and surveillance imaging are warranted to allow early detection of disease recurrence and/or metastasis.

### Acknowledgements

The authors would like to thank Associate Professor R. Max Conway, Dr. Geoff Whitehouse, and Dr Tricia Saurine for their contribution in the management of the patient.

### Financial Support and Sponsorship

None.

### Conflicts of interest

None.
